# Validating a network hub in leukaemia stem cells

**DOI:** 10.18632/oncoscience.339

**Published:** 2017-02-24

**Authors:** Lisa E. Hopcroft, Sheela A. Abraham, Tessa L. Holyoake

**Affiliations:** Paul O'Gorman Leukaemia Research Centre, Institute of Cancer Sciences, University of Glasgow, Gartnavel General Hospital, Glasgow G12 0YN, UK

**Keywords:** chronic myeloid leukaemia, stem cells, network analysis, transcriptional regulation

In our 2016 paper in Nature, we demonstrated that the transcription factors (TFs) p53 and c-MYC comprise a dual signaling “hub” in Chronic Myeloid Leukaemia (CML) leukaemic stem cells (LSC)[1]. This finding was derived from differential expression and network analyses of several proteomic and transcriptomic datasets describing CML LSC and normal stem/progenitor cells (SPC). Perturbation of this dual hub using gene knockdown strategies and/or specific drugs targeting the hub was shown to induce selective kill of LSC (the primitive stem cells that are responsible for initiating and sustaining the disease [2]). Importantly no such effects were observed on normal SPC, across a range of experimental *in vitro* models, and two *in vivo* murine models of CML.

It was essential to validate the *in silico* CML network analysis that identified the two TFs as therapeutic targets. Specifically, we sought to address the concern that, as p53 and c-MYC are a tumour suppressor and oncogene, respectively, we would expect to find them as hubs in network analyses of any oncogenic data. Furthermore, our observation could theoretically be compounded by literature bias, given that p53 and c-MYC are extensively studied in biological research and therefore over-represented in resources used to build interaction networks. Here, we seek to describe our approach to these concerns in more detail.

Our candidate network of 58 proteins emerged from (1) an analysis of isobaric-tag mass spectrometry (MS) data measuring relative protein expression in SPC from CML patients (n=3) and healthy donors (n=2), and (2) a subsequent network analysis (using MetaCore) to identify important regulators of the CML LSC specific proteomic signature. As mentioned above, p53 and c-MYC emerged as the two primary hubs (see Figure 1(a) in [1]).

**Figure 1:**
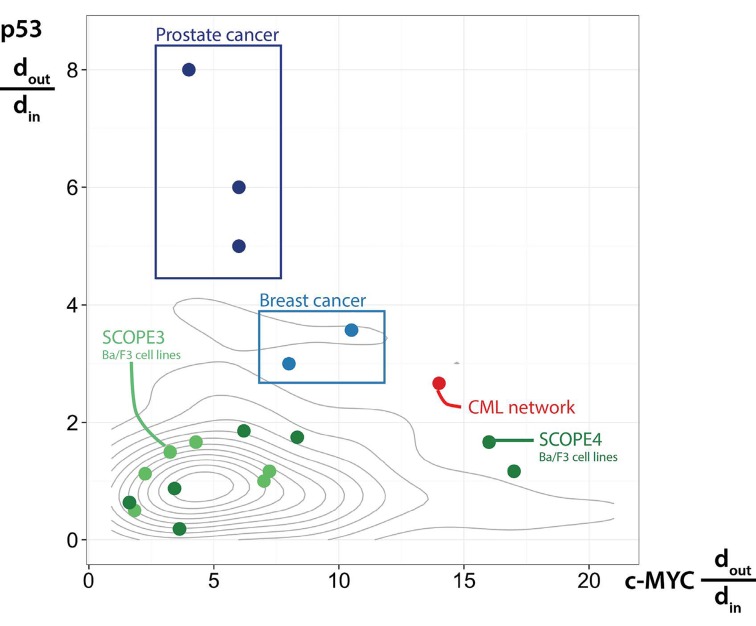
Validating a dual hub network topology Plotting the ratio of outgoing (d_out_) to incoming (d_in_) connections to understand the role of p53 and c-MYC in each network. Other primary datasets shown in blue, cell line datasets shown in green, random networks summarised by grey contours and our CML network shown in red.

To ensure that this process was not susceptible to literature bias, we subjected 50 random sets of proteins (drawn from the >4000 proteins observed in the MS experiments) to the same network analysis. The connectivity of p53 and c-MYC within these random networks was then determined. Critical to our hypothesis, viable pharmacological target nodes need to be (i) densely connected to and (ii) lie upstream of signaling pathways. In order to compare our observations in the CML network with other networks, we calculated a topological bias statistic (d_out_/d_*in*_), to quantify the ratio of outgoing to incoming connections for each protein. This value will increase as the number of outgoing connections exceeds the number of incoming connections; therefore high ratios indicate predominantly upstream connectivity. With an understanding of how this ratio varies for both p53 and c-MYC in randomly generated data sets of proteins, we were able to assess whether the topological ratios for p53 and c-MYC observed in our CML network were likely to have occurred by chance. Figure 1 shows that our CML network (red) fell outwith the random network distributions (summarised by grey contours), with only 2/50 random networks demonstrating equivalent or greater upstream bias (p=0.04) for p53 and c-MYC. Interestingly, the topological analyses of two proteomic datasets derived from Ba/F3 cell lines expressing leukaemogenic protein tyrosine kinases (TK; e.g., Flt3-ITD, Fip1L/PDGFRα) fell largely within the region occupied by the random protein sets, including the Ba/F3 cell line transduced with the BCR-ABL1 oncogene [3]. We interpreted this to suggest, firstly, that malignancies driven by constitutively active protein TK may not share aberrant signaling networks (at least not those that depend on p53 and/or c-MYC) and, secondly, that cell line models are limited in their replication of the signaling pathways found in primary material, such as CML LSC. Ultimately, these results demonstrate that there was no evidence of a confounding literature bias in our methodology.

To assess the second point (whether our dual hub was observable in other malignant diseases), we repeated the analysis in other datasets comparing primary, human cancer cells to their closest normal counterparts. Optimising MS technologies to assay these cells is technically challenging and as such, very few datasets exist that compare to our study and to which we could apply our methods. We were able to identify two such datasets, capturing proteomic expression in two clinical subtypes of breast cancer [4] and three clinical subtypes of prostate cancer [5]. The topological ratios for p53 and c-MYC in the context of breast (light blue) and prostate (dark blue) cancer are shown alongside those of the CML network, the random protein sets and the previously analysed cell line datasets (Figure 1). Unlike the cell line data, these primary cell results fall largely outwith the random distributions, reinforcing the idea that cell lines are limited in their ability to replicate signaling pathways found in primary cells. It is also clear that the prostate cancer network showed only a dominant p53 bias (with MTA and CREB1 emerging as more dominantly upstream), whilst the breast cancer network demonstrated a dual, but weaker p53/c-MYC signature, as compared to CML (here, a stronger upstream bias was observed for CREB1, TIF1-beta and coagulation factor XIIIA).

This work corroborates our original finding that p53 and c-MYC have dominant roles in CML signaling, which was substantiated in the subsequent laboratory validation [1]. This work represents a novel paradigm in which an unbiased systems biology approach performed on primary patient SPC has uncovered a novel therapeutic drug combination. This work also represents an innovative clinical intervention for CML and as a result, we are actively progressing a drug combination targeting the hub towards clinical trial.
